# Individual and community-level factors associated with home birth: a mixed effects regression analysis of 2017–2018 Benin demographic and health survey

**DOI:** 10.1186/s12884-021-04014-x

**Published:** 2021-08-11

**Authors:** Francis Appiah, Bernard Afriyie Owusu, Josephine Akua Ackah, Patience Ansomah Ayerakwah, Vincent Bio Bediako, Edward Kwabena Ameyaw

**Affiliations:** 1 Department of Population and Health, University of Cape Coast, Cape Coast, Ghana; 2 Berekum College of Education, Berekum, Bono Region Ghana; 3 Department of Optometry, University of Cape Coast, Cape Coast, Ghana; 4 Faculty of Health, School of Public Health, University of Technology Sydney, Sydney, Australia

**Keywords:** Individual level factors, Community-level factors, Home birth, Maternal health, Public health, Benin

## Abstract

**Background:**

Home birth is a common contributor to maternal and neonatal deaths particularly in low and middle-income countries (LMICs). We generally refer to home births as all births that occurred at the home setting. In Benin, home birth is phenomenal among some category of women. We therefore analysed individual and community-level factors influencing home birth in Benin.

**Methods:**

Data was extracted from the 2017–2018 Benin Demographic and Health Survey females’ file. The survey used stratified sampling technique to recruit 15,928 women aged 15–49. This study was restricted to 7758 women in their reproductive age who had complete data. The outcome variable was home birth among women. A mixed effect regression analysis was performed using 18 individual and community level explanatory variables. Alpha threshold was fixed at 0.05 confidence interval (CI). All analyses were done using STATA (v14.0). The results were presented in adjusted odds ratios (AORs).

**Results:**

We found that 14% (*n* = 1099) of the respondents delivered at home. The odds of home births was high among cohabiting women compared with the married [AOR = 1.57, CI = 1.21–2.04] and women at parity 5 or more compared with those at parity 1–2 [AOR = 1.29, CI = 1.01–1.66]. The odds declined among the richest [AOR = 0.07, CI = 0.02–0.24], and those with formal education compared with those without formal education [AOR = 0.71, CI = 0.54–0.93]. Similarly, it was less probable for women whose partners had formal education relative to those whose partners had no formal education [AOR = 0.62, CI = 0.49–0.79]. The tendency of home birth was low for women who did not have problem in getting permission to seek medical care [AOR = 0.62, CI = 0.50–0.77], had access to mass media [AOR = 0.78, CI = 0.60–0.99], attained the recommended ANC visits [AOR = 0.33, CI = 0.18–0.63], belonged to a community of high literacy level [AOR = 0.24, CI = 0.14–0.41], and those from communities of high socio-economic status (SES) [AOR = 0.25, CI = 0.14–0.46].

**Conclusion:**

The significant predictors of home birth are wealth status, education, marital status, parity, partner’s education, access to mass media, getting permission to go for medical care, ANC visit, community literacy level and community SES. To achieve maternal and child health related goals including SDG 3 and 10, the government of Benin and all stakeholders must prioritise these factors in their quest to promote facility-based delivery.

**Supplementary Information:**

The online version contains supplementary material available at 10.1186/s12884-021-04014-x.

## Background

Maternal death is higher for women in sub-Saharan Africa and Southern Asia where 86% of the global maternal deaths occur [39]. Globally, there have been significant progress in the decline of maternal mortality ratio (MMR) from 342 deaths to 211 deaths per 100,000 live births [32] but this has been disproportionate. For instance, compared to maternal deaths in high-income countries (11 per 100,000 livebirths), 462 deaths per 100,000 live births occur in low income countries such as Benin [36]. In Benin, MMR is 405 per 100,000 livebirths, and this is unexpectedly high. Although the global call for universal health coverage and skilled birth attendance has not been homogeneously achieved across regions, the higher MMR in low-income countries reflects several factors. These include inequalities in access to quality health services, distance to healthcare facilities, deep-rooted sociocultural beliefs [37] and several practices including poor-healthcare seeking behaviour and home care delivery [2, 22].

The benefits of healthcare delivery/childbirth in the prevention of maternal deaths cannot be overemphasized, yet, a considerable number of women continue to give birth at home [30]. Whereas planned births may be attended at home by a skilled birth attendant in Benin, at the home environment, childbirth is more likely to be assisted by TBAs, relatives and other unregistered health practitioners [14, 41]. This is particularly true for women from poorer backgrounds in Benin where about 40% of them give birth at home [32]. The period of pregnancy has constantly proven to be life threatening for women and their unborn children. Compared to skilled birth attendance which is commonly provided in healthcare facilities, and associated with lower rates of adverse maternal and birth outcomes [5, 9, 27]. Home birth put the health of mothers and their babies at increased risk of maternal and neonatal morbidities, mortality and disability as they are more likely to miss expert care and the appropriate environment during delivery [10]. About 75% of pregnancy complications including antepartum and postpartum haemorrhage, infections, puerperal complications, preeclampsia and eclampsia, and unsafe abortions usually coincide with home births [36]. Consequently, the WHO recommends that all births should be overseen by a skilled birth attendant [15, 38].

Over the years, a number of interventions have been instituted at both global and national levels, to boost uptake of institutional deliveries. Such initiatives notably include the Sustainable Development Goal 3 target 3.1, which seeks to reduce global maternal mortality ratio to less than 70 per 100,000 livebirths. At the national level, the government of Benin implemented a five-year health strategy (2015–2020) with the aim of reducing preventable deaths among vulnerable populations in the country [33]. Among the activities in the strategy is the enhanced extension of maternal and child health services to resource-limited areas by increasing community healthcare workers. While these initiatives are essential, barriers to utilising quality maternal health service must be identified and addressed at both health system and societal levels [36].

Several direct and indirect causes of maternal mortality have been identified and the latter includes home birth [19]. As reported by Lerberg et al. [19], a wide difference exists between women’s preference of place of delivery and the actual venue for birth and this has negative consequences for both the mothers and their babies. In this paper, we analysed the 2017–2018 Demographic and Health Survey data of Benin to determine both individual and community level factors predicting home births. The paper seeks to answer this question: What are the individual and community level factors that determine home births among women in Benin? Understanding the predictors of home birth is of high relevance to both local and public health understanding of home birth, as well as highlighting areas where national interventions will yield a cost-effective result. To our knowledge, this is the first mixed effect regression analysis of nationally representative large-scale data on predictors of home births in Benin.

## Materials and methods

### Extraction of data for the study

The present study made use of the women’s file of the 2017–2018 Benin Demographic and Health Survey (BDHS). The 2017–2018 BDHS was conducted in order to better operationalize and monitor the indicators of the Sustainable Development Goals (SDGs) for Benin. The National Institute of Statistics and Economic Analysis (INSAE) [23] carried out the survey in collaboration with the Ministry of Health. Assistance was obtained from Inner City Fund (ICF) through the international DHS (The Demographic and Health Survey) Program. The government of the Republic of Benin and the Agency of the United States for International Development (USAID) funded the 2017–2018 BDHS. The survey took place from November 6, 2017 to February 28, 2018. The main issues captured include fertility, fertility and infant and child mortality, contraceptive use, maternal health, children’s health, vaccination and other essential issues. The survey used a stratified sampling technique that was representative nationally. The 2017–2018 BDHS involved 14,156 households. Specifically, all women aged 15–49 in selected households and were present the night before the survey were eligible to be interviewed. This led to 16,233 eligible women, however 15,928 completed the interviews at a response rate of 98.1%. The current study was restricted to 7758 women aged 15–49 who had complete data. The dataset is publicly available at Measure DHS repository (https://dhsprogram.com/data/dataset/Benin_Standard-DHS_2017.cfm?flag=1) and details of the sampling processes are available in the 2017–2018 BDHS report [13].

### Description of study variables

#### Outcome variable

The main outcome variable for the study was “home birth among women aged 15-49”. In the 2017–2018 BDHS, women were asked where they gave birth during their last childbirth which was posed as “Where did you deliver [name]?” accompanied by these responses: “home”, “other home”, “government hospital”, “government health centre/clinic”, “government health post/Community-based Health Planning and Services (CHPS)”, “other public”, private hospital/clinic”, “maternity homes”, and “others”. Following previous study [1], these responses were grouped into two responses and are “home birth” to denote every delivery that occurred outside health facility setting and “health facility delivery” to signify those that delivered in a health facility. “Home birth” was recoded as “1″ whereas “health facility delivery” was recoded as “0″.

#### Explanatory variables

Eighteen explanatory variables were selected for the study. These are age, wealth status, religion, education, marital status, total children ever born, occupation, partner’s education, access to mass media, getting medical help for self; getting permission to go, getting medical help for help: getting money needed for treatment, getting medical help for self: distance to health facility, ANC visit and health decision making. All these constituted the individual-level factors. The community variables comprised sex of household head, community literacy level and community socioeconomic status. For clarity of presentation, some of the explanatory variables were recoded. Age was recoded as “19 years and under”, “20–34 years” and “35 years and above”. Religion was recoded as “Non-religious” and “Religious.” Education was recoded into “Without formal education” and “Formal education”; marital status was recoded into “Never married”, “Married”, “Cohabiting”, “Widowed” and “Divorced”; considering fertility rate of Benin which is about 5.7 children per woman [13], total children ever born was recoded into “1–2 births”, “3–4 births”, and “5 births or more”. Occupation was also recoded as “Not working” and “Working”, partner’s education recoded into “Without formal education” and “Formal education”. Access to mass media was constructed from three prime variables: frequency of reading newspaper/magazine; frequency of listening to the radio; and frequency of watching television. Each of these media variables had three responses: ‘not at all’, ‘less than once a week’, and ‘at least once a week’. A composite variable was created whereby those that indicated ‘less than once a week’ and ‘at least once a week’ were categorised as having access to mass media whilst ‘not at all’ was considered as not having access to mass media. ANC visit was recoded into “Below recommended” for less than eight visits and “recommended” for at least eight ANC visits, health decision making was recoded into “Alone”, “Respondent and partner” and “Others”. Community literacy level was generated by decomposing community literacy into three categories: “Low”, “Medium” and “High” and similar procedure was followed to generate community socioeconomic status. All these variables were selected due to their theoretical significance to maternal healthcare utilisation, specifically home delivery [1, 43].

#### Statistical analysis

The study set forth to unravel individual and community-level factors that determine home birth among Benin women aged 15–49. Based on this aim, these procedures were followed to analyse the dataset. The weighting factor built in the dataset (v005/100000) and the “svy command” were applied to deal with over and under sampling biases and to gauge for the complex survey design and generalizability of the findings respectively. The proportion of women who delivered home or otherwise were calculated. This was followed with univariate descriptive computation of the explanatory variables to show the summary statistics of the data. Thereafter, a cross-tabulation computation of outcome variable across the explanatory variables was done and the results were presented in proportions and percentages. Additionally, a chi square test of independence was applied to assess the association between the outcome variable and the explanatory variables at 0.05 alpha threshold. The variance inflation factor (VIF) command was applied to interrogate the collinearity among the explanatory variables and the results (Additional file 1) showed no evidence of multicollinearity between them (Mean VIF = 1.50, maximum VIF = 2.44, minimum VIF = 1.04).

At 95% confidence interval, four regression models were built. The first model was a null model (Model 0) and accounted for the variations in home births, which is attributable to the clustering of the primary sampling units (PSUs) without the effect of both individual and community-level factors. In the DHS, primary sampling units are equivalent to clusters or communities that houses a number of households [11]. Therefore, in this study, we considered clustering in the PSUs to be the same as clustering across communities. The second model (Model I) considered individual-level factors solely whereas the third model (Model II) considered the effects of community-level factors on home births alone. Finally, the last model (Model III) was a full model containing both individual and community-level factors. The results for the fixed effects were presented as adjusted odds ratio (AOR) whereby any odds less than one was interpreted as reduced likelihood to home births whilst an odds higher than 1 meant otherwise. Since the models were nested, the Akaike information criterion (AIC) and Bayesian information criterion (BIC) techniques were used to measure their fitness [1, 43]. The random effects which are measures of variation of home births across communities or clusters, were expressed in terms of Intra-Class Correlation (ICC) [1, 28, 43]. These were calculated to quantify the degree of variation of home delivery across clusters and the proportion of variance explained by successive models. The analyses were done using STATA version 14.0.

##### Ethical considerations

The present study made use of an already existing dataset. Hence, authors of this article were not involved in the implementation of the original study. However, the request to use the dataset was sought from Measure DHS. Measure DHS assessed the intent of our request and subsequently granted us access to download the dataset. The dataset is available at the Measure DHS repository at https://dhsprogram.com/data/dataset/Benin_Standard-DHS_2017.cfm?flag=1. Measure DHS anonymised the dataset before making it available for public use. The 2017–2018 BDHS reported that all ethical considerations applicable to human research participation were followed. Details of the ethical considerations are available in the survey report [13].

## Results

### Descriptive results for the study

It was found that 14% (1099) of the women delivered at home (data not shown). Also, home births was high among the poorest (35%), those who were not affiliated to any religion (27%) and women without formal education (19%). Additionally, 14% among the married delivered at home and was same among the cohabiting (14%). There were no observations for never married, widowed and divorced. Women who had given birth to 1–2 children (11%) dominated in home births. Home births was prevalent among women who were not working (18%), those whose partners had no formal education (22%) and women who had no access to mass media (18%). It was also phenomenal among those who declared that getting permission to seek medical help for self was problematic (26%), just as those who revealed that getting money needed for treatment was difficult (16%). Also, a significant proportion of women who revealed that distance to health facility was a challenge delivered at home (24%) as displayed in Table 1.

**Table 1:** Descriptive results for the study (*N* = 7758)

Study variables	Weighted (N)	Weighted (%)	Home births (Weighted %)	X^2^(*p*-value)
**Individual-level factors**				
***Age***				3.3133(0.191)
≤ 19	388	5	16	
20–34	5446	70	14	
≥ 35	1924	25	15	
***Wealth status***				1.0e+ 03(0.000)
Poorest	1671	22	35	
Poorer	1606	21	17	
Middle	1589	20	11	
Richer	1535	20	4	
Richest	1357	17	0.4	
***Religion***				56.5548(0.000)
Religious	7329	94	13	
Non-religious	429	6	27	
***Education***				343.6895(0.000)
Without formal education	5155	66	19	
Formal education	2603	34	4	
***Marital status***				5.6783(0.017)
Married	6132	79	14	
Cohabiting	1626	21	14	
***Total children ever born***				74.8063(0.000)
1–2 births	2762	35	11	
3–4 births	2465	32	10	
≥ 5	2531	33	6	
***Occupation***				21.3699(0.000)
Not working	1324	17	18	
Working	6434	83	13	
***Partner’s education***				500.2258(0.000)
Without formal education	4296	55	22	
Formal education	3462	45	4	
***Access to mass media***				200.3966(0.000)
No	5410	70	18	
Yes	2348	30	6	
***Getting medical help for self: getting permission to go***				261.6552(0.000)
Big problem	1920	25	26	
Not a big problem	5838	75	10	
***Getting medical help for self: getting money needed for treatment***				59.6779(0.000)
Big problem	4235	55	16	
Not a big problem	3523	45	11	
***Getting medical help for self: distance to health facility***				363.2372(0.000)
Big problem	2200	35	24	
Not a big problem	5058	65	9	
***ANC visits***				103.1457(0.000)
Below recommended	7056	91	15	
Recommended	702	9	1	
***Health decision making***				112.9952(0.000)
Alone	717	9	9	
Respondent and partner	2633	34	10	
Others	4408	57	18	
**Community-level factors**				
***Residence***				137.7570(0.000)
Urban	2903	37	9	
Rural	4855	63	18	
***Sex of household head***				42.4693(0.000)
Male	6731	87	15	
Female	1027	13	8	
***Community literacy level***				842.8920(0.000)
Low	2742	35	28	
Medium	2490	32	11	
High	2526	33	2	
***Community socioeconomic status***				773.1515(0.000)
Low	4083	53	24	
Medium	1169	15	6	
High	2506	32	1	

Source: 2017–18 BDHS

Women who did not meet the recommended number of ANC visits (15%) dominated the proportion that delivered at home. Also, 18% of those who depended on others for making health decisions had home births. Similarly, 18% rural residents delivered at home; similar to women whose household heads were males and delivered at home (15%). Significant proportion of women in communities with low literacy level (28%) and low socioeconomic status (24%) delivered at home (Table 1).

### Fixed effects result

The Model III shows the fixed effects result of home births. Relative to the poorest, the odds of home births was low among the richest [AOR = 0.07, CI = 0.02–0.24]. The tendency to deliver at home was low among those with formal education compared with those without formal education [AOR = 0.71, CI = 0.54–0.93]. The probability to patronise home births increased among the cohabiting as compared with the married [AOR = 1.57, CI = 1.21–2.04]. Women who had given birth to 5 or more children were more probable to deliver at home as compared to those with 1–2 children [AOR = 1.29, CI = 1.01–1.66]. Those whose partners had formal education were less likely to deliver at home relative to those whose partners had no formal education [AOR = 0.62, CI = 0.49–0.79]. Also, those who had access to mass media had lower likelihood of home births as compared with those without access to mass media [AOR = 0.78, CI = 0.60–0.99]. Women who mentioned that getting permission to seek medical help for self was unproblematic had lower likelihood to deliver at home compared with those who viewed it as problematic [AOR = 0.62, CI = 0.50–0.77]. It was evident that those who attained the recommended ANC visits were less likely to deliver at home compared with those who did not meet the recommended number of ANC visits [AOR = 0.33, CI = 0.18–0.63]. Women belonging to communities of high literacy level had lower odds to deliver at home [AOR = 0.24, CI = 0.14–0.41], just as among those from communities of high socioeconomic status compared to those from communities of low socioeconomic status [AOR = 0.25, CI = 0.14–0.46] (Table 2).

**Table 2:** Mixed effects of maternal and community-level factors and home births

Independent variables	Model 0	Model I	Model II	Model III
	aOR[95%CI]	aOR[95%CI]	aOR[95%CI]	aOR[95%CI]
**Fixed effect results**				
**Individual-level factors**				
***Age***				
≤ 19		Ref		Ref
20–34		1.08[0.74–1.58]		1.10[0.75–1.61]
≥ 35		1.12[0.72–1.75]		1.19[0.76–1.85]
***Wealth status***				
Poorest		Ref		Ref
Poorer		0.65***[0.53–0.81]		0.68***[0.55–0.84]
Middle		0.53***[0.42–0.69]		0.61***[0.47–0.78]
Richer		0.29***[0.20–0.41]		0.42***[0.29–0.61]
Richest		0.22***[0.01–0.07]		0.07***[0.02–0.24]
***Religion***				
Religious		Ref		Ref
Non-religious		1.38*[1.02–1.86]		1.33[0.98–1.79]
***Education***				
Without formal education		Ref		Ref
Formal education		0.63**[0.48–0.82]		0.71*[0.54–0.93]
***Marital status***				
Married		Ref		Ref
Cohabiting		1.49**[1.14–1.93]		1.57**[1.21–2.04]
***Total children ever born***				
1–2		Ref		Ref
3–4		1.16[0.93–1.45]		1.19[0.95–1.49]
≥ 5		1.28*[1.00–1.62]		1.29*[1.01–1.66]
***Occupation***				
Not working		Ref		Ref
Working		0.92[0.73–1.15]		0.92[0.74–1.15]
***Partner’s education***				
Without formal education		Ref		Ref
Formal education		0.57***[0.44–0.72]		0.62***[0.49–0.79]
***Access to mass media***				
No		Ref		Ref
Yes		0.78*[0.60–0.99]		0.78*[0.60–0.99]
***Getting medical help for self: getting permission to go***				
Big problem		Ref		Ref
Not a big problem		0.59***[0.47–0.73]		0.62***[0.50–0.77]
***Getting medical help for self: getting money needed for treatment***				
Big problem		Ref		Ref
Not a big problem		1.12[0.89–1.41]		1.08[0.86–1.36]
***Getting medical help for self: distance to health facility***				
Big problem		Ref		Ref
Not a big problem		0.84[0.67–1.06]		0.92[0.74–1.15]
***ANC visit***				
Below recommended		Ref		Ref
Recommended		0.32***[0.17–0.60]		0.33**[0.18–0.63]
***Health decision making***				
Alone		Ref		Ref
Respondent and partner		1.03[0.72–1.47]		0.98[0.68–1.40]
Others		1.39[0.99–1.95]		1.29[0.92--1.80]
**Community-level factors**				
***Residence***				
Urban			Ref	Ref
Rural			0.97[0.62–1.50]	0.94[0.63–1.41]
***Sex of Household Head***				
Male			Ref	Ref
Female			0.89[0.66–1.20]	0.85[0.62–1.15]
***Community literacy level***				
Low			Ref	Ref
Medium			0.29***[0.19–0.45]	0.37***[0.24–0.55]
High			0.12***[0.71–0.22]	0.24***[0.14–0.41]
***Community socioeconomic status***				
Low			Ref	Ref
Medium			0.28***[0.16–0.49]	0.37***[0.22–0.63]
High			0.08***[0.05–0.15]	0.25***[0.14–0.46]
**Random effect results**				
PSU Variance[95%CI]	5.45[4.24–7.00]	2.19[1.66–2.88]	2.25[1.73–2.93]	1.73[1.30–2.30]
ICC	0.62	0.40	0.41	0.34
LR Test	1653.79***	498.95***	682.12***	399.42***
Wald X^2^	Ref	278.62***	222.05***	375.94***
**Model specification analysis**				
Log likelihood	−2412.8451	− 2230.3118	− 2288.2112	−2178.3287
AIC	4829.69	4504.624	4592.422	4412.657
BIC	4843.603	4657.666	4648.074	4607.439

*aOR* adjusted Odds Ratio, *CI* Confidence Interval in square brackets, *Ref* Reference Category; *p < 0.05, **p < 0.01, ***p < 0.001

### Random effect results

Results of the random effect show that variability existed with regards to home births [σ^2^ = 5.45, CI = 4.24–7.00]. It was found that about 62% of the variability observed in the null model was attributable to variability in the intra class correlation (ICC) characteristics (ICC = 0.62). More so, the ICC values reduced in model I (ICC = 0.40), model II (ICC = 0.41) and in model III (ICC = 0.34). This implies that the variability to deliver at home is attributable to the variability in the primary sampling units (PSUs) (Table 2).

From the model specification analysis, the ideal value of the estimated coefficient of the null model was lower (log likelihood = − 2412.8451), but it improved in the succeeding models, particularly in model III (log likelihood = − 2178.3287). In the same vein, the null model was less appropriate (Akaike Information Criteria [AIC] = 4829.69, Bayesian Information Criteria [BIC] = 4843.603). However, there were substantial improvement in the desirability of the models specifically in model III (AIC = 4412.657, BIC = 4607.439). Therefore, model III is well specified relative to the other models. The fitted models are summarised on Table 2 below.

## Discussion

The quest to ensure improved maternal health has prompted the need for several interventions and programs of which facility-based delivery is notable. This study investigated maternal and community factors associated with home births in Benin. As found in this study, 14% of women delivered at home; a marginal increase from the 13% reported in 2012 [13]. This may imply that socio-cultural and economic factors influencing women’s choice of delivery place might not have changed within the past 6 years (between 2011 and 2012 and 2017–2018). The findings from our study reveal several individual and community level factors that influence the chance of women to give birth at home.

The results showed that relative to the poorest, the odds to utilise home births declined among the richest. This finding echoes the effect of economic inequalities on women’s health care utilisation [12]. Over the years, support from the Benin government in the form of fee exceptions for natural and caesarean section deliveries has contributed to increasing maternal health care utilization across the country [18]. Meanwhile, there is also a concern for other costs which are not covered under such exemptions. For instance, Lange et al. [18] reported that women still pay for care either in form of under-the-table payments to health workers or prescribed payments for medications and procedures not covered by the exemptions. Since women in richer households are characterized with financial convenience, they are able to absorb all costs associated with transportation and facility-based delivery services and are therefore, less likely to deliver at home. Women from poor households, however, are usually affected by financial challenges and this tend to increase their chances of delivering at home. The results are consistent with other studies in Africa and beyond [7, 8, 16, 21, 41]. Yaya [40] recommended that to address this inequality, the advocacy and interventions to enhance women’s economic empowerment should be strengthened.

The study revealed a negative association between education and home birth whereby women who had a formal education or stayed with a partner with a formal education had lesser odds to utilise home births. Similar findings had been reported in Ghana [8], Kenya [17, 21], and Gabon [40] that since women with higher education have increased health information, they desire high quality health care and are less likely to choose the home as a place of delivery. For instance, Mekonnen and Mekonnen [20] reported that education enhances women’s autonomy from which they develop the confidence and capabilities to make decisions regarding their own health; thus the decision to use health facilities for safe deliveries.

Congruent to other studies [6, 8, 34], parity was significantly associated with women’s chances for home birth. Women who had given birth to 5 or more children were most probable to deliver at home compared to those with 1–2 children. Tsegay, et al. [31], however, found the association between parity and women’s place of delivery to be a product of chance. Furthermore, regarding marital status, the probability to deliver at home increased among the cohabiting as compared with the married. This could be explained by the fact that women who conceive out of wedlock are less motivated to use institutionalised health services due to community stigmatisation and marginalisation [20] as well as discriminatory attitudes of health providers.

Women who had access to mass media had lower likelihood to utilise home births as compared to those without access to mass media. The mass media exposes individuals to a wide range of health information which, in effect, influences their health decisions [35]. The more desirous a woman finds information about institution-based delivery from various mass media sources, the less likely she would want to deliver at home. From our study, the odds reduced by 24%. This is congruent with the findings of some studies from low-and-middle income countries [3, 4] that conclusively advocate the importance of mass media as a major strategy in promoting the benefits of health facility delivery. In Eritrea however, Kifle and colleagues found that not all mass media is important in determining women’s choice of delivery place. Print media (reading newspapers) was significant while the rest (TV, radio) were trivial. They explained that women who read newspapers are likely to be educated and receptive of health information, therefore, their low preference for home deliveries. Similarly, Yaya et al. [41] indicated that access to media was important, yet with significant results for only watching TV and listening to the radio.

Women that did not find getting permission to seek medical help for self as problematic were less likely to deliver at home compared to those that viewed it as problematic. When it is difficult for women to be granted permission to seek medical help for themselves, it results in delay, and this may accrue to their delivering at home and vice versa. This affirms a study in Ghana by Sumankuuro et al. [29] who narrated that women’s lack of autonomy to seek care without prior permission from husbands or other family relatives delayed the timely use of skilled care and health facility resources. Montagu et al. [22] acknowledged the interplay of intra-familial decision-making power and its interference with women’s desire to seek institution-based health care irrespective of their wealth status. In their study, despite their wealth status, women who could not deliver at health facilities explained that their preference were deemed “not necessary” by a household decision maker.

Furthermore, it was evident that those that attained the recommended ANC number of visits were less likely to deliver at home compared with those that could not meet the recommended number of ANC visits. Our findings corroborate those from previous studies that demonstrate the positive effects of antenatal care in reducing home deliveries and enhancing institutional deliveries [7, 25, 26, 42]. Women who visit the recommended ANCs have better contact with skilled providers, access to information about danger signs during pregnancy as well as the importance of facility delivery [24]. Therefore, the desire to deliver at home wanes as preference for facility-based delivery increases.

Our study is novel in exploring community level factors (literacy levels and socioeconomic status) and its impact on home births. Most studies have either looked at literacy and socio-economic standing at the individual or household level [3, 7, 12, 34, 40] and not necessarily at the community level. We found that women belonging to communities with high literacy levels and socio-economic status had lower odds of delivering at home. The influence of community characteristics such as literacy and socioeconomic status on women’s individual choice to deliver at home may operate largely through latent social relationships. More specifically, if a woman is found in a community where her social circle are literate and socioeconomically enhanced with a higher tendency to deliver at health facilities, she may easily be influenced to follow same behavioural practice. To some extent, even receive the needed assistance which may be in the form of health information and financial support. Additionally, such communities are more likely to have collective understanding of health information and the essence of facility-based delivery. They are therefore in a better position to lobby for or contribute to constructing health facilities to address the maternal needs of women in the community; in effect, addressing physical barriers.

Methodologically, we accounted for the hierarchical structures embedded in the sampling design of Benin’s Demographic and Health Survey and found that there are inequalities at the community level. This is reminiscent of important socio-cultural and economic characteristics that makes one community different from the other and thereby indicate a call for community specific interventions in addressing the inequalities associated with place of delivery.

### Strength and weakness

This study provides substantial contribution to policy debates and research on home births in Benin. The study used a nationally representative sample size for the analysis, and for that matter the findings are generalizable to women aged 15–49 in Benin. It is worthy to note that this study is not without some limitations. First, although the current BDHS dataset was used, this study is cross-sectional in nature and do not allow causal inference between maternal and community factors and home births. Also, the BDHS does not provide information on the type or skills of birth attendants at home births, however most births at home in Benin are expected to be attended by unskilled birth attendants. Thus, it is not possible to generalize our findings to other contexts where home births are commonly assisted by skilled birth attendants. Given that socio-cultural practices, expectations and patriarchy influence issues of fertility, women may feel uncomfortable providing accurate information. For instance, some of them might misreport the permission from their partners to choose a place of birth.

## Conclusion

Given the dangers associated with unskilled birth attendance and/or inadequate screening of risk factors/comorbidities that characterises home-based births in low resource settings, stringent interventions need to be put in place to encourage facility-based delivery while considering potential influencing factors. Factors that have significant association with home births are wealth status, education, marital status, parity, partner’s education, access to mass media, getting permission to go for medical care, ANC visit, community literacy level and community socioeconomic status. To achieve MCH-related goals including SDG 3 and 10, the government of Benin and other stakeholders must prioritise these factors in their quest to promote facility-based delivery in Benin.

## Supplementary Information

**Additional file 1.** Multicollinearity test results.

## Data Availability

The datasets generated and/or analysed during the current study is publicly available in the Measure DHS repository at https://dhsprogram.com/data/dataset/Benin_Standard-DHS_2017.cfm?flag=1.
