# Supplementary material for: Individual and community-level factors associated with home birth: a mixed effects regression analysis of 2017–2018 Benin demographic and health survey
Source: BMC Pregnancy Childbirth. 2021 Aug 11;21:547. doi: 10.1186/s12884-021-04014-x (PMC8359262; doi:10.1186/s12884-021-04014-x)
Supplement: Supplementary file 1 — Additional file 1. Multicollinearity test results. [file 12884_2021_4014_MOESM1_ESM.docx]

**Multicollinearity Testing**

**Appendix 1: Multicollinearity test results**

| Study variables | VIF | 1/VIF |
| --- | --- | --- |
| Community socioeconomic status | 2.44 | 0.409982 |
| Wealth status | 2.36 | 0.423920 |
| Total children ever born | 1.99 | 0.501554 |
| Age | 1.91 | 0.522443 |
| Community literacy level | 1.80 | 0.556741 |
| Education | 1.67 | 0.597409 |
| Partner’s education | 1.64 | 0.610929 |
| Getting medical help for self: distance to health facility | 1.52 | 0.658546 |
| Getting medical help for self: getting money needed for treatment | 1.39 | 0.718190 |
| Getting medical help for self: getting permission to go | 1.38 | 0.724946 |
| Residence | 1.30 | 0.771941 |
| Access to mass media | 1.29 | 0.775398 |
| Health decision making | 1.09 | 0.913392 |
| ANC visit | 1.09 | 0.920726 |
| Occupation | 1.09 | 0.921339 |
| Religion | 1.04 | 0.958494 |
| Sex of Household Head | 1.04 | 0.960949 |
| Marital status | 1.04 | 0.965357 |
